# Influenza in Africa

**DOI:** 10.1371/journal.pmed.1000182

**Published:** 2009-12-15

**Authors:** Maria Yazdanbakhsh, Peter G. Kremsner

**Affiliations:** 1 Department of Parasitology, Leiden University Medical Center, Leiden, The Netherlands; 2 Medical Research Unit, Albert Schweitzer Hospital, Lambarene, Gabon; 3 Institute for Tropical Medicine, University of Tübingen, Tübingen, Germany

## Abstract

Maria Yazdanbakhsh and Peter Kremsner argue that there needs to be better awareness, surveillance, and clinical management of common febrile diseases in Africa, especially influenza.

**Summary Points**

- Influenza activity displays a seasonal pattern in temperate areas with marked peaks in the winter, but influenza is present all year round throughout the tropics.
- A well-established network of surveillance systems, World Health Organization (WHO) Flu Net, is in place in Europe and North America, providing continuous data on influenza burden and spread of viral types and subtypes.
- Recent threats of pandemic influenza are prompting the establishment of active monitoring in parts of Southeast Asia and Latin America.
- But prevalence and incidence of influenza in most tropical countries, especially in Africa, are largely unknown, and improved surveillance is needed.
- Similarly, little information on influenza vaccine efficacy in tropical Africa is available, and clinical trials are needed.

## How Little We Know

Whereas in Europe and North America most of the influenza cases are reported between December and March, in tropical and subtropical regions such as in Brazil [1] or in Hong Kong [2] cases are seen throughout the year. Epidemic peaks in the tropical areas mostly occur in between those found in the Northern and Southern hemispheres. A recent survey over 7 years in Brazil showed that annual peaks of influenza cases occurred in association with the rainy seasons [3]. Important reports on spatial and temporal data that describe the global circulation of influenza highlight the fact that there is virtually no data from Africa [4],[5]. Indeed, until recently, the burden of influenza in Africa was believed to be negligible. However, sporadic reports from the Gambia [6], Senegal [7], Congo [8], Madagascar [8], Kenya [9], Ivory Coast [10], and from Gabon [11], have indicated that influenza is circulating and may be causing epidemics regularly. The study in Gabon recorded extremely high levels of antibodies to influenza A H3N2 virus in schoolchildren [11]. The haemagglutination inhibition (HI) antibodies to this influenza A virus at titers of 1,530 (ranging from 80 to 17,920) indicated that the virus had been circulating within the community in the recent past. In addition, almost all children, had anti-H1N1 HI titers above 40, while 40% showed antibodies to influenza B with HI titers of 40 or above, again highlighting the fact that multiple influenza virus strains are present in the region. The recent swine flu pandemic provides an interesting example. In the WHO influenza A (H1N1 swine flu) update of May 2009, many countries, but none in Africa, reported virus victims [12]; whereas two reports appeared in October 2009 that showed data on confirmed swine flu cases from South Africa [13] and Kenya [14], indicating that the virus was circulating in Africa, but because of the lack of a rigorous surveillance system, it was not reported as readily.

## Influenza and Other Febrile Diseases

Clinically, influenza is not distinguishable from most other infectious diseases with fever in the tropics. In this context malaria is of particular interest when considering the African continent. In tropical Africa, malaria is an important infectious disease and is still thought to be the main cause of febrile episodes in children. However, the threshold of clinical manifestation of malaria is strongly influenced by the endemicity of *Plasmodium falciparum* infection in an area: in very low transmission areas, any microscopically detectable parasitemia would indicate malaria, whereas in regions of high transmission, a certain parasitemia needs to be reached to lead to malaria at least from 5 years of age on. Recent, mainly unpublished observations show that there is a considerable drop in malaria incidence [15] and in *P. falciparum* prevalence rate [16] in some African countries. Despite this reduction, the old habit of treating every child with fever with antimalarials continues. As fever due to many infectious diseases wanes after a few days without treatment, the belief that medical staff are successfully treating malaria cases lingers on.

A recent study in Lambarene, Gabon, illustrates the extent of the problem and the unresolved conundrum. In and around Lambarene all febrile children are still treated with antimalarials, mainly amodiaquine-artesunate [17]. In a study of 1,000 consecutive children presenting with fever at our research center in Lambarene, the results from the thick blood smears indicated that only about 5% had malaria. Recent serological tests have shown that parts of the febrile illnesses in Lambarene are due not only to influenza, but also to dengue fever, chikungunya disease, and streptococcal pneumonia [18]. In addition, in Tanzania where malaria is considered to be highly endemic, D'Acremont and coworkers refer to recent data indicating that only 10%–40% of under-5-year-old patients with fever have malarial parasites in rural areas [19]. Thus, in Lambarene and perhaps elsewhere in Africa, the majority of febrile cases may be unnecessarily exposed to antimalarial drugs, with the well-recognized negative consequences.

It is acknowledged that the problem of diagnosing influenza-like illness is already challenging in resource-rich settings apparent from data collected on the ongoing pandemic of influenza A (H1N1, swine flu). Examining symptomatic individuals with recent history of travel to countries where the H1N1 virus was circulating indicated that other respiratory viruses such as rhinovirus, coronavirus, or para-influenzavirus were responsible for influenza-like illness [20]. Therefore, not surprisingly, yet often ignored, there is simultaneous transmission of different respiratory viruses and bacteria in addition to malaria that lead to febrile illnesses in Africa and elsewhere in the tropics.

The task of diagnosing and treating febrile illnesses properly in resource-poor tropical settings is daunting. Yet, with attention to upgrading clinical research in Africa focused on combatting the well known diseases such as AIDS, tuberculosis, and malaria, we need to start taking a few steps towards implementing programs that deal with influenza-like illness. Thus in each of the Northern, Western, Eastern, Central, and Southern African regions one well-established research center could be identified to act as a surveillance center. Already in Senegal and South Africa such centers exist, but there is a need for identifying new ones in other regions and upgrading and intensifying activities in already existing ones. Training should ensure that epidemiological data can be gathered on attack rates, clinical spectrum of illness, and risk factors, while molecular diagnosis of collected samples confirms influenza and identifies the strain/subtypes. In close collaboration with WHO centers, the behavior of the influenza virus would then be monitored properly on the African continent. Contrary to common belief, excellent clinical research centers are developing in Africa with good epidemiologists, information technology infrastructure, and laboratory equipment.

## Influenza Vaccine

There is no information on influenza vaccine efficacy in tropical Africa. The question of whether the immune system of populations living in tropical African environments would react similarly to a vaccine developed mainly for populations restricted to certain geographical areas of the world needs to be considered. Helminthic infections, malaria, and other chronic parasitic infections along with nutritional status lead to altered functioning of the immune system [11]. Not only Th2 responses [21] but also regulatory T cells are expanded during many parasitic infections [21],[22] and are thought to affect responses to unrelated antigens. Interestingly, a study of meningococcal vaccination of infants in Ghana showed lower titres of MenA and MenC bactericidal antibodies than in other studies in Africa [23].

In a recent trial in Gabon, schoolchildren from a rural and a semi-urban setting were vaccinated with influenza vaccine [11]. Clear immunological differences were seen in response to the vaccine in rural versus semi-urban children. Weaker Th1 and pro-inflammatory cytokine responses to influenza virus antigens were seen in vaccinated rural children compared to the semi-urban vacinees. Antibody levels following vaccination increased in all, but to a different extent in subjects from rural than in semi-urban areas. The antibody response following vaccination to A-H1N1 and B virus strains were significantly lower in rural compared to urban schoolchildren. The clinical relevance of such findings remains unanswered, even 30 years after studies of McGregor in the Gambia examining influenza-like illness and reporting differences in antibody reactivity in African populations and populations of European descent [24].

Therefore it is important to undertake clinical trials with this vaccine in different regions of Africa. The end point in these studies should be the occurrence of influenza cases to assess the efficacy of the vaccine in Africa and learn about a threshold for a protective HI titer.

## Concluding Remarks

Taken together, data from sporadic studies suggest that influenza is prevalent in Africa and the disease may have considerable impact on morbidity and mortality on the continent. A raised awareness of the presence of common febrile diseases such as influenza is essential for the clinical management of patients. To this end proper surveillance systems should be set up in already existing and well-established clinical research centers to understand the epidemiology of influenza in Africa, which in turn may help the processes of decision making regarding influenza vaccination on the continent, which may have a high impact on health in Africa.

## Abbreviations

HI: haemagglutination inhibition
